# Cell Surface Sialylation and Fucosylation Are Regulated by L1 via Phospholipase Cγ and Cooperate to Modulate Neurite Outgrowth, Cell Survival and Migration

**DOI:** 10.1371/journal.pone.0003841

**Published:** 2008-12-02

**Authors:** Ya-li Li, Guang-zhi Wu, Gavin S. Dawe, Li Zeng, Shu-sen Cui, Gabriele Loers, Thomas Tilling, Li Sun, Melitta Schachner, Zhi-Cheng Xiao

**Affiliations:** 1 Department of Anatomy, Research Centre of Heart, Brain, Hormone and Healthy Aging, and the State Laboratory of Brain and Cognitive Science, Li Ka Shing Faculty of Medicine, The University of Hong Kong, Hong Kong, China; 2 Department of Clinical Research, Singapore General Hospital, Singapore, Singapore; 3 Department of Pharmacology, Yong Loo Lin School of Medicine, National University of Singapore, Singapore, Singapore; 4 Surgery Department, China-Japan Union Hospital, Jilin University, Changchun, China; 5 Developmental Biology Division, Institute of Molecular and Cell Biology, Singapore, Singapore; 6 Neurodegeneration Research Laboratory, National Neuroscience Institute, Singapore, Singapore; 7 Zentrum für Molekulare Neurobiologie, University of Hamburg, Hamburg, Germany; 8 Keck Center for Collaborative Neuroscience, Rutgers University, Piscataway, New Jersey, United States of America; Tufts University, United States of America

## Abstract

**Background:**

Cell surface glycosylation patterns are markers of cell type and status. However, the mechanisms regulating surface glycosylation patterns remain unknown.

**Methodology/Principal Findings:**

Using a panel of carbohydrate surface markers, we have shown that cell surface sialylation and fucosylation were downregulated in *L1^−/y^* neurons versus *L1^+/y^* neurons. Consistently, mRNA levels of sialyltransferase ST6Gal1, and fucosyltransferase FUT9 were significantly reduced in *L1^−/y^* neurons. Moreover, treatment of *L1^+/y^* neurons with L1 antibodies, triggering signal transduction downstream of L1, led to an increase in cell surface sialylation and fucosylation compared to rat IgG-treated cells. ShRNAs for both ST6Gal1 and FUT9 blocked L1 antibody-mediated enhancement of neurite outgrowth, cell survival and migration. A phospholipase Cγ (PLCγ) inhibitor and shRNA, as well as an Erk inhibitor, reduced ST6Gal1 and FUT9 mRNA levels and inhibited effects of L1 on neurite outgrowth and cell survival.

**Conclusions:**

Neuronal surface sialylation and fucosylation are regulated via PLCγ by L1, modulating neurite outgrowth, cell survival and migration.

## Introduction

Glycosylation of proteins and lipids is a prime example of a cellular process that is not under the direct control of the genome. This contributes to the functional diversity required to generate extensive phenotypes from a limited genotype [1]. Glycosylation is a crucial post- or co-translational modification of more than 50% of all eukaryotic proteins according to database analyses [2]. It is affected by a multitude of factors, such as cellular metabolism and the rate of cell growth. Accumulated evidence indicates that glycan structures play important roles in various contexts, including differentiation, development, fertilization, inflammation, and cell–cell recognition [3], [4]. Glycosylation defects in mice and their links to the development of diseases have shown that the mammalian glycome contains a significant amount of biological information [5], [6]. Moreover, defects in glycosylation pathways are often associated with psychomotor/mental retardation or other neuropathological symptoms as seen in most congenital diseases of glycosylation [7]. It is believed that specific glycosylation patterns are expressed in a cell type-specific and developmentally regulated manner. Thus, identification of the molecular mechanisms underlying regulation of glycan diversity will help to elucidate how an ensemble of glycans displayed at the cell surface governs signal transduction and cell–cell communication via multivalent interactions with proteins.

Fucose is one of the most important glycans expressed at the cell surface. It is a deoxyhexose that is present in a wide variety of organisms. In mammals, fucosylated carbohydrate structures have important roles in a variety of biological and pathological processes, such as tissue development, angiogenesis, fertilization, selectin-mediated leukocyte-endothelial adhesion, inflammation, host immune response, and tumor metastasis, including Notch receptor family signaling events [8]. Alterations in the expression of fucosylated oligosaccharides occur in several pathological processes, including cancer and atherosclerosis [8]. Fucosylated glycans are generated by fucosyltransferases (FucTs) that are responsible for the catalysis of fucose transfer from the donor guanosine-diphosphate fucose (GDP-fucose) to various acceptor molecules including oligosaccharides, glycoproteins, and glycolipids. During early organ development, compartment formation outside the nervous system is determined by carbohydrate-dependent signal transduction between cell surface recognition molecules as elegantly shown for Notch and its cell surface binding partners Jagged/Serrate and Delta. Ligand-receptor interaction between these molecules is determined by the O-fucose-β1,3-N-acetylglucosaminyl-transferase, Fringe, which determines the Notch-bearing cell's reaction to its binding partners [10]. These examples highlight the importance of carbohydrates in cell–cell interactions outside the nervous system.

Another very important monosaccharide is sialic acid. Sialic acids are expressed as terminal sugars with a shared nine-carbon backbone in several classes of cell surface and secreted glycan molecules [4]. Sialic acids provide negative charge and hydrophilicity to vertebrate cell surfaces, mask subterminal galactose residues from recognition by certain receptors, and act as receptors for pathogens and toxins [4], In particular, sialic acids play an important role during mammalian development [11]. In the nervous system, polysialic acid is nearly exclusively carried by the neural cell adhesion molecule (NCAM), a protein belonging to the immunoglobulin (Ig) superfamily. Polysialylated NCAM is involved in the development of the nervous system, *N*-methyl-D-aspartate (NMDA) receptor-dependent synaptic plasticity, and regeneration in the adult [7]. Several vertebrate proteins have been found that mediate specific recognition events involving sialic acids [4], such as the cell recognition molecule L1, another Ig superfamily member, that specifically recognizes 2,3-linked sialic acids present on the heavily glycosylated cell surface protein, CD24 [7], [12].

L1 itself is one of many carbohydrate-carrying molecules in the nervous system, where it is widely expressed and involved in many aspects of neural development, regeneration and synaptic plasticity in the adult [13]. Various human genetic disorders with prominent nervous system defects are caused by mutations in L1 [14]. Cell recognition molecules at the cell surface are not only donors, but also acceptors of carbohydrates. These carbohydrates mediate interactions between recognition molecules in *cis* or *trans* and thereby modulate their functions as receptors at the cell surface and as signal transducers [7]. Modulation of these interactions occurs through finely tuned synthesis of glycan chains depending on the neural cell type and its developmental state. However, it is unknown whether these cell recognition molecules are also involved in regulation of glycan diversity at the cell surface.

Lectins are proteins which recognize specific glycan structures. Due to this property, they have been extremely useful in studying glycan variation [15], [16]. Glycans and lectins usually interact with lower affinities than those found for protein-protein interactions. However, lectin-glycan interactions are characterized by a significant avidity given that most lectins can bind multiple glycan moieties and do so with considerable specificity. Taking advantage of this, in the present study, we have used lectins and carbohydrate-specific antibodies to investigate the changes in glycosylation patterns on cell surfaces of neurons stimulated with L1 antibodies. We have demonstrated that L1 plays a role in modulating both sialylation and fucosylation at cell surfaces through increased expression of both ST6Gal1 and FUT9 via a phospholipase Cγ-mediated mechanism by which it enhances neurite outgrowth, cell survival and migration of neurons.

## Materials and Methods

### Antibodies, lectins and inhibitors

Goat polyclonal anti-mouse, rat and human FUT9 antibodies were purchased from Santa Cruz Biotechnology, Inc. (Santa Cruz, CA, USA). Mouse monoclonal anti-human ST6Gal1 antibody was purchased from Chemicon International, Inc. (Temecula, CA, USA). Rabbit polyclonal anti-mouse, rat and human PLCγ antibodies were purchased from Cell Signaling Technology, Inc. (Danvers, MA, USA). The monoclonal anti-mouse L1 antibody 557 was produced and purified as previously described [17]. L3, L4 and L5 antibodies were produced as previously described [18], [19], [20], [21]. Biotinylated lectins were purchased from Vector Laboratories, Inc. (Burlingame, CA, USA). R-Phycoerythin (R-PE)-conjugated mouse anti-rat monoclonal, PerCP-CY5.5-conjugated rat anti-mouse IgM monoclonal and R-PE-conjugated rat anti-mouse IgM monoclonal antibodies were purchased from BD Biosciences (Franklin Lakes, NJ, USA). Inhibitors were either from Calbiochem, San Diego, CA, USA (U73122, Cdc25 phosphatase inhibitor II, and Erk inhibitor) or from Sigma, St. Louis, MO, USA (LY294002 and KT5720).

### Mice

For primary culture of cerebellar granule neurons, we used L1-deficient mice (129x1SvJL1/tTA L1^−/y^, abbreviated line name: L1ki129) and wild-type littermates (129x1SvJL1/tTA L1^+/y^). In L1 deficient mice, the expression of L1 was completely abolished by insertion of a tetracycline-controlled transactivator into the second exon of the L1 gene [22]. L1^−/y^ mice and L1^+/y^ mice were generated from the mating of 129x1SvJ males and L1ki129 heterozygous females. The experiments involving animals were approved by the Institutional Animal Care and Use Committee (IACUC) of Singapore General Hospital.

### Cell culture

All cell culture reagents were purchased from Invitrogen Life Technologies (Merelbeke, Belgium) unless indicated otherwise. Dissociated cerebellar granule neurons were obtained by sedimenting trypsinized cerebellar cells from 6- to 8-day-old mice through a Percoll gradient [23]. The neuronal cell suspension was seeded on poly-L-lysine (Sigma; Mr 70,000–150,000)-coated culture dishes and maintained in chemically defined serum-free medium containing basal medium Eagle's supplemented with 2 mM L-glutamine, 1 mg/mL bovine serum albumin, 12.5 µg/mL insulin, 4 nM thyroxine, 100 µg/mL transferrin, 30 nM sodium selenite, 0.1 mg/mL streptomycin and 10 U/mL penicillin in a 5% CO_2_ incubator at 37°C. The monoclonal L1 antibody was produced and purified as previously described [17]. Non-immune rat IgG served as a negative control. After treating with L1 antibody or rat IgG (control) for 24 h, neurons were dissociated with 0.05% trypsin/0.04% EDTA to single cells for assay by flow cytometry. NIH3T3 cells were maintained in DMEM supplemented with 10% FBS, 0.5 U/mL penicillin, 0.5 U/mL streptomycin. Cells were passaged every 2 or 3 days using 0.05% trypsin/0.04% EDTA. The single cells obtained were used for assay by flow cytometry.

### Flow cytometric analysis of surface expression of carbohydrates

Cell surface carbohydrate expression was assessed by indirect immunofluorescence detection using a flow cytometer, FACSCalibur (Becton-Dickinson, San Jose, CA, USA) equipped with an argon laser with an emission wavelength at 488 nm. CellQuest Pro software (Becton-Dickinson) was used for cell acquisition and analysis. Cerebellar neurons were prepared and cultured as described above. The cells were digested with 0.05% trypsin and washed twice with PBS (Gibco, Long Island, NY, USA). Single cell suspension was prepared in PBS with 10% FBS with the concentration adjusted to 10^7^ cells/ml for indirect antibody labeling. Antibody labelings of the cells were carried out using 4 ml sterile tubes (Falcon, Becton-Dickisnon). In brief, 50 µl of cell suspension (5.0×10^5^ cells) was aliquoted into 4 ml sterile centrifuge tubes (Falcon) and incubated with primary antibody or lectin (1 µg) targeting one of 14 cell surface carbohydrates in PBS with 10% FBS for 30 minutes at 4°C in the darkness. Cells were washed 3 times with chilled PBS and then incubated with secondary antibody conjugated with R-phycoerythin or Peridinin chlorophyll protein (R-PE or Per-CP, 1 µg) in 50 µl of PBS with 10% FBS for a further 30 minutes at 4°C in the dark. Cells were washed 3 times with chilled PBS, fixed in PBS with 1% formaldehyde (Sigma, St. Louis, USA) and kept at 4°C in darkness before flow cytometric analysis. 10^4^ cells from each tube were acquired for analysis. Unstained cells and cells incubated with secondary antibody alone were used as controls for autofluorescence.

### Microarray analysis

Procedures for cDNA synthesis, labeling, and hybridization were carried out in accordance with the manufacturer's protocol (Affymetrix Inc., Santa Clara, CA, USA). All experiments were performed using Affymetrix mouse genome Genechip 430 2.0 array and MOE 430 A array. Briefly, 15 µg total RNA was used for first strand cDNA synthesis with a high-performance liquid chromatography purified T7-(dT) 24 primer. Synthesis of biotin labeled cRNA was carried out using the ENZO RNA transcript labeling kit (Affymetrix). For hybridization, 15 µg fragmented cRNA was incubated with the chip in 200 µl hybridization solution in a Hybridization Oven 640 (Affymetrix) at 45°C for 16 hours. Genechips were washed and stained with streptavidin-phycoerythrin using the microfluidic workstation™ (Affymetrix) and scanned with Affymetrix Microarray scanner system. Data were analyzed using Spotfire software (Somerville, MA, USA).

### Real-time PCR

To compare the transcript levels, total RNA from neurons was isolated using an RNeasy mini kit (Qiagen, Valencia, CA, USA). Total RNA concentration was determined using a spectrophotometer to measure absorptions at 260 nm and 280 nm wavelengths. To check RNA integrity, total RNA was separated in a 1% agarose gel containing formaldehyde and stained with ethidium bromide. Reverse transcription was performed with the SuperScript™ First-strand Synthesis System for RT-PCR Kit (Invitrogen, Carlsbad, CA), in accordance with the manufacturer's protocol. Briefly, 5 µg total RNA was reverse transcribed with random hexamers. First-strand reverse transcribed cDNA was then diluted 1∶10 in water before use for real-time PCR. cDNA was subjected to real-time PCR using the 7000 ABI Detection System (Applied Biosystems, Foster City, CA, USA). Real-time PCR reactions (Applied Biosystems, California, USA) included 1× TaqMan Universal PCR master mix, 1× Assays-on-Demand TM Gene Expression Assay Mix, and 2 µl of template in a 20 µl reaction volume. The following program was run: 50°C for 30 min, 95°C for 15 min, then 40 cycles of 94°C for 15 sec, 60°C for 1 min, followed by plate reading. The assay ID number of Fut9 is Mm01223272_m1; the assay ID number of St6gal1 is Mm00486119_m1. Gapdh was used as a reference, and its assay ID number is Mn99999915_g1.

### Neurite outgrowth assay

Diethyl ether-cleaned glass coverslips were coated overnight at 4°C with poly-L-lysine (100 µg/mL in distilled water) and washed three times with distilled water. Purified cerebellar granule neurons from cerebella of 6- to 8-day-old mice were seeded onto the coverslips (1×10^5^cells/mL) and allowed one hour to adhere. Subsequently, different concentrations of anti-L1 antibody 557 or equimolar amounts of rat IgG were added. After cultivation for 24 hours, the cells were fixed for one hour by adding 4% paraformaldehyde and stained with Coomassie brilliant blue (0.2%) for 30 minutes at room temperature. Coomassie brilliant blue-stained cultures were analyzed by evaluating the length of individual neurites. Only neurites that did not contact other cells or neurites and had a length of at least one cell diameter were measured [14]. For each cell measured, the total length of all neurites and the length of the longest neurite were determined. For each experimental condition, at least three independent experiments were performed in duplicate and more than 120 neurites were measured (30 neurites/coverslip) with LSM Image Browser System. Non-immune rat IgG treatment was used as a negative control.

### Migration assay

Costar transwell polycarbonate filters (5.0 µm pore size) were used in the migration assay [24]. The undersurfaces of the 6.5 mm transwell membranes were coated with anti-L1 antibody in PBS (200 µg/ml) overnight at 4°C; rat IgG coating was used as a negative control. After coating, filters were blocked with 2% BSA in PBS for one hour. Subsequently, 2.5×10^5^ cerebellar granule cells/ml were plated in culture medium into the upper chamber and allowed to migrate through the pores onto the coated undersurfaces at 37°C in a CO_2_ incubator. After 24 hours, cells from the inner surface of the insert were gently wiped out with cotton-tipped swabs, and the inserts were fixed with 4% paraformaldehyde for 30 minutes and stained with 0.2% crystal violet solution in PBS for 30 minutes. After a final PBS washing, the cells were examined under an inverted-TS100 microscope (Nikon Singapore Pte Ltd, Singapore) to confirm proper morphology, and the dye was extracted with 10% acetic acid in PBS. The absorbance was measured at 570 nm using a microplate reader (BioTek Instruments, Inc., Winooski, VT, USA). Dye levels are directly proportional to the numbers of cells. Data are presented as mean±S.E.M.

### Cell survival assay (MTT assay)

1×10^5^ cerebellar granule cells were cultured in 96 well plates for 24 hours. Afterwards, 10 µl 5 mg/ml MTT solution was then added and cells were cultured at 37°C for 4 hours. After washing with PBS, 20% SDS in PBS was added to lyse the cells and dissolve the dye crystals. The OD (absorbance) at 570 nm was determined with an Elx800 universal microplate reader (BioTek Instruments) and the percentage of surviving cells was calculated [25].

### mRNA knockdown using short hairpin RNAs (shRNAs)

Cerebellar granule neurons from cerebella of 6- to 8-day-old mice were isolated. (3–4)×10^6^ isolated neurons were combined with 1 µg GFP plasmid, 1 µg respective shRNA vector or an empty control vector (control group) and 100 µl neuron-specific Nucleofector solution (Amaxa, Cologne, Germany), then transferred to an Amaxa-certified cuvette. Eletroporation was performed according to the manufacturer's Amaxa Nucleofection protocol with the Amaxa Nucleofector™ II device and mouse neuron O-005 program [26]. After transfection cells were transferred into culture dishes using the culture medium described above. Twenty-four hours later, cells were fixed with 4% paraformaldehyde. Subsequently, the cells were used for immunocytochemical staining or neurite length measurement. For NIH-3T3 cell transfection, 100 µl cell line-specific Nucleofector solution (Amaxa) and NIH3T3 cells were mixed and transfected with the Amaxa Nucleofector™ II device as described for the transfection of neurons using the U-030 program. For culturing, DMEM medium was used as described above.

### Western blot analysis

Transfected NIH3T3 cells or cerebellar neurons were lysed with RIPA buffer (1% NP-40, 0.5% sodium deoxycholate, 0.25% SDS, 0.1 5 M NaCl, 0.01 M sodium phosphate, 2 mM EDTA, 1 mM NaF, 50 mM sodium fluoride, 1 mM sodium orthovanadate, 0.5 mM PMSF, 10 µg/ml aproptinin and leupeptin) [27]. Protein concentration was determined by the Bio-Rad BCA assay. Samples containing equal amounts of protein were resolved by SDS-PAGE and transferred to polyvinylidene difluoride (PVDF) membranes (Millipore, Billerica, MA, USA) followed by incubation with primary and secondary antibodies and chemiluminescent detection using the ECL kit (GE Healthcare Bio-Sciences AB, Uppsala, Sweden). The following antibodies were used in this study: FUT9 and ST6Gal1 (Santa Cruz Biotechnology), PLCγ1 (Cell Signaling Technology), and β-actin (Sigma).

### Immunocytochemical staining

After transfection by electroporation [26], cultured cells were grown on PLL-coated coverslips. After 24 hours in culture, cells were fixed with 4% paraformaldehyde and permeabilized with 0.1% Triton X-100 for 30 minutes, after washing with PBS, fixed and permeabilized cells were incubated with 5% BSA for 1 hour, then incubated with the primary antibody for 2 hours and with the secondary antibody for 1 hour. After drying, the cells were mounted with Hard Set™ fluorescence mounting medium (Vector Laboratories, Inc. Burlingame, CA, USA) and observed under a Leica microscope.

### Data analysis

All data were expressed as mean±S.E.M. Statistical evaluations were achieved by one-way analysis of variance and Student's *t* test. Differences were considered to be significant when p<0.05.

## Results

### Glycosylation patterns are changed on the surface of *L1^+/y^* and *L1^−/y^* neurons

**Figure 1:**
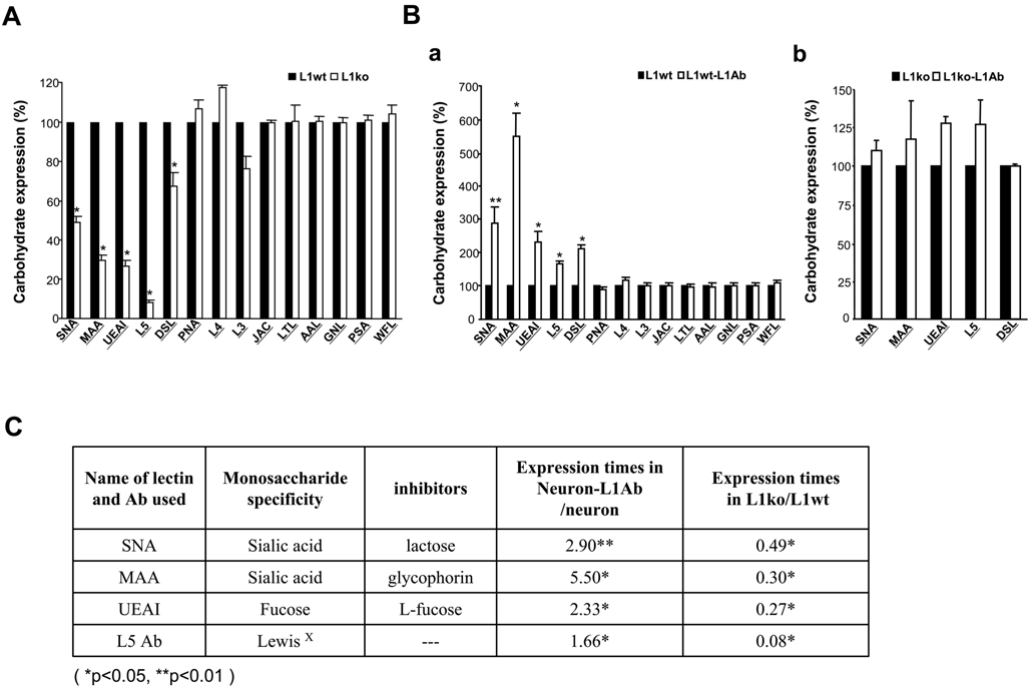
Glycosylation patterns on the cell surface of L1^+/y^ and L1^−/y^ neurons with and without treatment with anti-L1 antibodies. A. Neurons isolated from L1^+/y^ (L1wt) and L1^−/y^ (L1ko) mice were subjected to flow cytometry analysis using a panel of carbohydrate surface markers, including lectins and antibodies against carbohydrates. The quantitative result showed that the expression of carbohydrates recognized by SNA, MAA, UEAI, and DSL as well as L5 antibody was significantly decreased in the neurons of L1^−/y^ mice versus L1^+/y^ mice. B. After treatment with anti-L1 antibodies, neurons isolated from L1^+/y^ (L1wt-L1Ab) and L1^−/y^ (L1ko-L1Ab) mice were assayed by flow cytometry using a panel of carbohydrate surface markers, including lectins and antibodies against carbohydrates. The quantitative result showed that the expression of carbohydrates recognized by SNA, MAA, UEAI, DSL, and L5 antibody was significantly increased in L1wt-L1Ab neurons versus neurons treated with rat IgG only (“L1wt”). (a). The quantitative result showed that the expression of carbohydrates recognized by SNA, MAA, UEAI, DSL, and L5 antibody was no significant different in L1ko-L1Ab neurons compared to L1^−/y^ neurons treated with rat IgG only (“L1ko”) (b). C. The expression patterns of carbohydrates were compared between the L1^+/y^ neurons treated with anti-L1 antibodies and L1^+/y^ neurons treated with rat IgG (neuron-L1Ab/neuron) and from neurons isolated from L1^−/y^ versus L1^+/y^ mice (L1ko/L1wt). L1 or L1 antibodies commonly regulated the sialylation and fucosylation on the cell surface of neurons. *: p<0.05, and **: p<0.001 by Student's test.

Since the cell adhesion molecule L1 is one of the important carbohydrate-carrying molecules at the neuronal cell surface and mediates interactions between other adhesion molecules in nervous system, we hypothesized that L1 might also modulate specific glycosylation patterns at neuronal cell surfaces. To test this hypothesis, carbohydrate expression at the cell surface of neurons isolated from *L1^+/y^* (wt) and *L1^−/y^* (ko) mice (the knockout of L1 is shown in Fig. S1) was assayed by flow cytometry. Cerebellar granule neurons from early postnatal brains were chosen for analysis, since L1 is highly expressed during early postnatal development and its expression peaks at postnatal day 7. A panel of carbohydrate surface markers, including lectins and antibodies against carbohydrates, such as the oligomannose-specific antibodies L3, L4 and the Lewis^X^ specific antibody L5 were used (Fig. S2). The quantitative results showed that the expression of α2,6-linked sialic acid (recognized by *Sambucus nigra* lectin; SNA), α2,3-linked sialic acid (recognized by *Maackia amurensis* lectin II; MAA), fucose (recognized by *Ulex europaeus* agglutinin I; UEAI), and *N*-acetyllactosamin in complex and hybrid sugars (recognized by Datura stramonium lectin; DSL) as well as Lewis^X^ (recognized by L5 antibody) were significantly downregulated in neurons of *L1^−/y^* mice compared with *L1^+/y^* neurons (Fig. 1A). These results suggest that L1 may modulate carbohydrate expression at neuronal cell surfaces.

### L1 regulates sialylation and fucosylation at cell surfaces through L1–L1 *trans*-interactions

L1 at the cell surface is not only a donor, but also an acceptor of certain carbohydrates, thereby forming complexes between apposing partner cell surfaces through *trans*-interactions [13]. L1 on one cell surface can interact homophilically with L1 on another cell surface and these homophilic L1–L1 *trans*-interactions can be mimicked by the treatment of L1 expressing cells with L1 antibodies [28]. To further confirm that activation of L1 is involved in the modulation of sialylation and fucosylation at cell surfaces, neurons isolated from *L1^+/y^* and *L1^−/y^* mice were cultured at a low density and treated with L1 antibodies for 24 hours. Then, using a panel of carbohydrate surface markers, we performed flow cytometry analysis. Notably, the expression of carbohydrates recognized by SNA, MAA, UEAI, DSL, and L5 antibody were significantly upregulated in the *L1^+/y^* (Fig. 1Ba), but not in the *L1^−/y^* (Fig. 1Bb) neurons after treatment with L1 antibodies compared with rat IgG treatment. In particular, the expression of α2,6-linked sialic acid was up-regulated 3-fold and the expression of α2,3-linked sialic was 6-fold increased in L1 treated wild-type neurons, but not in L1-deficient neurons. The expression patterns of carbohydrates regulated by L1 or L1 antibody are summarized in Fig. 1C. The presence of L1 as well as treatment with L1 antibodies had an impact on the expression of carbohydrates recognized by SNA, MAA, UEAI, and L5 antibodies on the cell surface of neurons. Both SNA and MAA lectins recognize terminal sialic acids with different linkages. UEAI recognizes terminal fucose, and the L5 antibody recognizes the fucose containing carbohydrate Lewis^X^. These results indicate that activation of L1 might regulate the sialylation and fucosylation patterns of cell surfaces glycoproteins and/or glycolipids.

### L1 regulates the expression of the sialyltransferase, ST6Gal1, and the fucosyltransferase, FUT9

**Figure 2:**
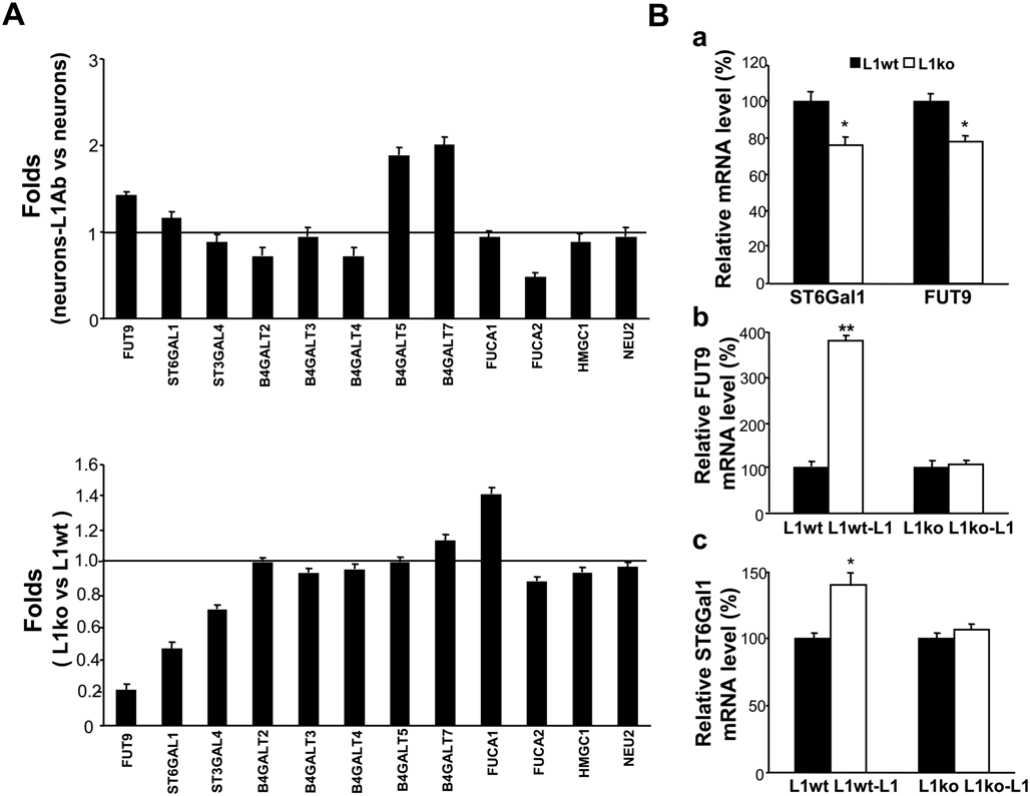
The mRNA expression of ST6Gal1 and FUT9 is modulated by L1. A. cDNA microarray analysis was performed to investigate the expression of genes involved in the synthesis and hydrolysis of the carbohydrates recognized by SNA, MAA, UEAI lectins and L5 antibodies by comparing between L1 antibody-treated neurons and rat IgG-treated neurons (“neurons-L1Ab” versus “neurons” group); and neurons isolated from the cerebellum of L1^−/y^ mice versus L1^+/y^ mice (“L1ko” versus “L1wt” group). Two microarrays per group were finished. The expression of fucosyltransferase 9 (FUT9) was elevated in the “neurons-L1Ab” versus “neurons” group (1.414-fold) and decreased in the L1ko neurons versus L1wt neurons group (0.218-fold), and β-galactoside-α2,6-sialyltransferase (ST6Gal1) expression was elevated in the “neurons-L1Ab” compared to the “neurons” group (1.249-fold) and decreased in the L1ko neurons versus L1wt neurons group (0.574-fold). B. To verify the results obtained by cDNA microarray analysis, quantitative RT-real-time PCR was performed. The expression of ST6Gal1 and FUT9 mRNAs was significantly downregulated in neurons isolated from L1^−/y^ (L1ko) versus L1^+/y^ (L1wt) mice (a) and upregulated in the L1-Fc-treated (“L1wt-L1”) versus Fc-treated (“L1wt”) group of L1^+/y^ neurons (b and c). No significant differences were observed in L1-Fc-treated (“L1ko-L1”) versus Fc-treated (“L1ko”) L1^−/y^ neurons (b and c).

To confirm that L1 is involved in the regulation of sialylation and fucosylation of cell surfaces, we investigated whether activation of L1 might alter the expression of specific sialyltransferases and/or fucosyltransferases. To identify the genes that respond to L1 activation, a cDNA microarray analysis was performed to compare L1 antibody-treated neurons with rat IgG-treated neurons (neurons-L1Ab versus control neurons group; Fig. 2A), and neurons isolated from the cerebellum of L1 ko mice with neurons from the cerebellum of L1 wt mice (L1 ko versus L1 wt; Fig. 2A). Of 27 genes related to synthesis and hydrolysis of carbohydrates examined, fucosyltransferase 9 (FUT9) was elevated in the L1Ab-treated neurons versus the IgG-treated control neurons (1.414-fold) and decreased in the L1 ko versus L1 wt group (0.218-fold); β-galactoside-α2,6-sialyltransferase (ST6Gal1) was elevated in the L1Ab-treated wt neurons, when compared to the IgG-treated wt neurons (1.249-fold) and decreased in the L1 ko versus L1 wt group (0.574-fold). Consistently, results of real-time PCR and cDNA microarray showed that both ST6Gal1 and FUT9 transcripts were significantly downregulated in *L1^−/y^* neurons versus *L1^+/y^* neurons (Fig. 2A and Ba). Notably, L1 antibody treatment led to a two-fold increase in the mRNA expression of UDP-Gal: βGlcNAc β1,4-galactosyltransferase (B4GALT5) and xylosylprotein β1,4-galactosyltransferase (B4GALT7) in wild-type neurons. In parallel, a 50% decrease in FUCA2 transcript levels could be observed. However, expression levels of the mRNAs encoding B4GALT5, B4GALT7 and FUCA2 were not changed in *L1^−/y^* neurons when compared with *L1^+/y^* neurons (Fig. 2A). Expression of the remaining genes was not significantly altered in both groups. Moreover, we treated both *L1^−/y^* neurons and *L1^+/y^* neurons with L1-Fc protein. Results of real-time PCR analysis showed that the mRNA expression levels of both ST6Gal1 and FUT9 were significantly upregulated (3.8- and 1.3-fold, respectively) in L1-treated *L1^+/y^* neurons versus Fc-treated *L1^+/y^* neurons, but there were no significant differences in L1-treated versus Fc-treated *L1^−/y^* neurons (Fig. 2Bb and c). Thus, these results demonstrate that activation of L1 by antibody treatment or homophilic interaction with L1-Fc upregulates the expression of both ST6Gal1 and FUT9 in neurons.

### Activated L1 promotes neurite outgrowth of neurons isolated from *L1^+/y^*, but not *L1^−/y^* mice

**Figure 3:**
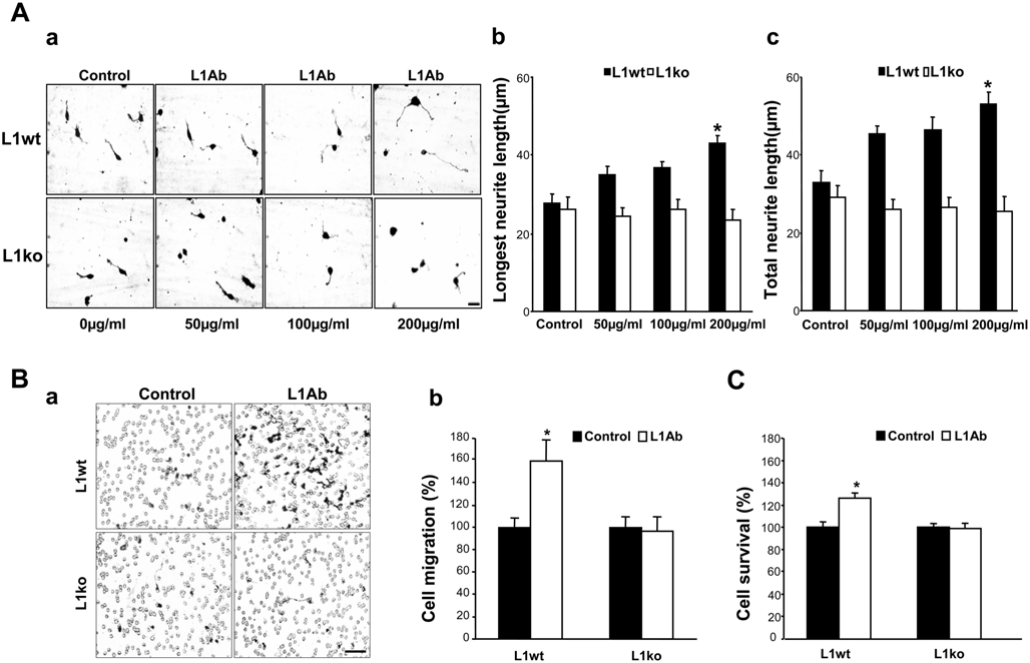
Activation of L1 promotes neurite outgrowth, cell migration and survival. A. Cerebellar granule neurons isolated from cerebella of 6- to 8-day-old L1^+/y^ (L1wt) and L1^−/y^ (L1ko) mice were seeded on coverslips coated with PLL. After 1 hour in culture, L1 antibody 557 or rat IgG (control) were added into the culture medium and the cells were cultured for a further 24 hours. a. Bright field micrographs of cerebellar neurons isolated from L1^+/y^ (L1wt) and L1^−/y^ (L1ko) mice and cultured with different concentrations of L1 antibodies (L1Ab: 50, 100, and 200 µg/ml). b. Longest neurite length. c. Total neurite length. B. To verify the role of L1 in cell migration, the undersurface of transwell membranes was coated with the L1 antibody. In the control group, the undersurface of transwell membranes was coated with rat IgG. Relative numbers of cells transmigrating through the membrane were determined by staining cells on the undersurface of the transwell membrane followed by cell lysis and measurement of the absorbance value. Optical density measures of the dye level were directly proportional to numbers of migrated cells. Neuronal migration induced by L1 antibody was significantly increased compared with neuronal migration induced by rat IgG in the neurons isolated from L1^+/y^ (L1wt) mice (O.D.: 0.294±0.024, 0.469±0.056, respectively), but not L1^−/y^ (L1ko) mice (O.D.: 0.248±0.024, 0.238±0.033, respectively). Photomicrographs illustrate the migrated cells on the undersurface of the membrane (a). Quantitative measurement results of the optical density of the dye recovered by cell lysis (b). C. To investigate the role of L1 in cell survival, MTT analysis was performed. After anti-L1 antibody (L1Ab) treatment (white bars), cell survival was significantly enhanced in the neurons isolated from L1^+/y^ (L1wt) mice (O.D.: 0.119±0.0047, 0.142±0.0055, respectively), but not L1^−/y^ (L1ko) mice (O.D.: 0.0938±0.0025, 0.0919±0.0046, respectively). Rat IgG treatment was used as control. *: p<0.05, by Student's test.

In agreement with our previous study [28], we further confirmed that L1 antibody treatment could mimic L1–L1 *trans* homophilic interactions to promote neurite outgrowth through a *trans*-interaction at cell surfaces (L1-Fc promoting neurite outgrowth is shown in Fig. S3). Neurons isolated from *L1^+/y^* and *L1^−/y^* mice were assayed for neurite outgrowth using different concentrations of L1 antibody 557 (Fig. 3Aa). Rat IgG treatment was used as a control. The length of the longest neurite and the total length of all neurites per cell were measured. Notably, L1 antibodies promoted neurite outgrowth of neurons isolated from *L1^+/y^* mice 1.5–2-fold, but not outgrowth of *L1^−/y^* neurons, both in terms of longest neurite (Fig. 3Ab) and of total neurite (Fig. 3Ac) lengths. Immunostaining result confirmed that the longest neurite is SMI312 positive (Fig. S4).

### Activated L1 promotes cell migration and survival of neurons

Moreover, to investigate whether L1-mediated neuronal survival and migration can be mimicked by L1 antibody treatment, we first determined the effect of L1 antibody 557 on migration of cerebellar neurons and neuronal survival. To investigate the role of L1 in cell migration, transwell membranes were coated with L1 antibody 557, which only stimulates the cells that express L1 at the cell surface. Transwell membranes were coated with rat IgG as a control. Cell migration was significantly increased by a factor of 1.5 in *L1^+/y^* neurons triggered by L1 antibodies relative to rat IgG-treated control cells, but not in *L1^−/y^* neurons (Fig. 3B). To examine whether L1 antibody treatment enhances cell survival, MTT analysis was performed. In agreement with the pro-survival effect of L1 demonstrated in our previous study [22], we showed that, after L1 antibody treatment, neuronal survival was enhanced by 30% in neurons isolated from *L1^+/y^*, but not *L1^−/y^*, mice (Fig. 3C). Rat IgG treatment was used as a control. Cell cycle analysis was performed as a complimentary experiment to further confirm the survival of neurons. An increment in S and G2/M phases, but no apoptosis, was observed in *L1^+/+^* neurons treated with L1Ab (Fig. S5).

### ShRNA-mediated knockdown of FUT9 and ST6Gal1 blocks L1-induced neurite outgrowth, cell migration and survival

**Figure 4:**
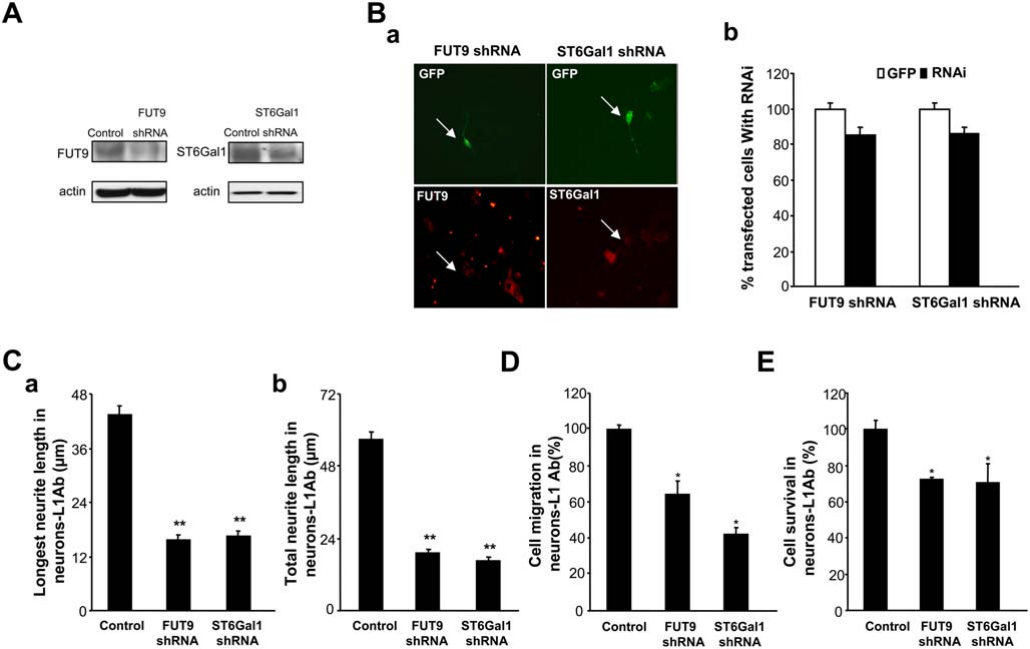
shRNAs of FUT9 and ST6Gal1 reduce L1 promoted neurite outgrowth, cell migration and survival. A. NIH3T3 cells were co-transfected with GFP plasmid plus empty vector (control) or shRNA of FUT9 or ST6Gal1 by electroporation. 24 hours later, cells were lysed and total protein was obtained. Western blotting was used to detect the expression of FUT9 and ST6Gal1. B. Neurons were dissected from the cerebellum of 6- to 8-day-old mice. Neurons were co-transfected with GFP plasmid plus shRNA of FUT9 or ST6Gal1 shRNA by electroporation and seeded on coverslips. GFP plasmid plus empty vector transfection was used as control. After 24 hours in culture, cells were fixed then stained with anti-FUT9 or anti-ST6Gal1 antibodies. (a): Immunocytochemistry for FUT9 or ST6Gal1 (red) in transfected neurons (green) indicated the knockdown efficiency, arrows indicate the same transfected cells in the upper and lower panels. (b): The co-transfection ratios of GFP and shRNA of FUT9 or ST6Gal1 to GFP and empty vector were quantified. C. Neurons were co-transfected with GFP plasmid plus empty vector (control) or shRNA of FUT9 or ST6Gal1, and seeded on coverslips coated with L1 antibody (L1Ab) or rat IgG. After 24 hours in culture, longest neurite length (a) and total neurite length (b) were measured. D. Cell migration assay was performed in L1antibody-treated neurons (Neurons-L1Ab) co-transfected with GFP plasmid plus empty vector (control) or shRNA of FUT9 or ST6Gal1. The percentage of cell migration was calculated by counting and comparing the number of green neurons on the upper surface and the undersurface of transwells. E. MTT analysis of L1antibody-treated neurons (Neurons-L1Ab) was performed after co-transfection with GFP plasmid plus empty vector (control, O.D.: 0.122±0.0054) or shRNA of FUT9 (O.D.: 0.0872±0.0011) or ST6Gal1 (O.D.: 0.0859±0.012). *: p<0.05; **: p<0.001, by Student's test, empty plasmid transfection neurons were controls.

To test the hypothesis that changes in sialylation and fucosylation contribute to L1-mediated neurite outgrowth, cell migration and survival, we performed the respective assays after knockdown of FUT9 and ST6Gal1 by RNA interference. To this end, we employed shRNAs of FUT9 and ST6Gal1, which could significantly reduce the expression of either FUT9 or ST6Gal1 in transfected NIH3T3 cells and neuron cells compared with empty vector-transfected cells (Fig. 4A and B). Both longest and total neurite lengths of L1 antibody treated neurons, but not untreated neurons were significantly reduced (about 70%) after transfection with either FUT9 shRNA or ST6Gal1 shRNA compared to control vector-transfected cells (Fig. 4Ca and b). Moreover, migration results also showed that the L1 antibody-induced increases in migration of neurons stimulated with L1 antibody were significantly decreased by 35% after transfection with FUT shRNA and 60% after transfection with ST6Gal1 shRNA when compared to mock-transfected neurons (Fig. 4D). Finally, survival of L1 antibody-triggered neurons was significantly reduced after transfection with these shRNAs (Fig. 4E). Together, these results demonstrate that sialylation and fucosylation may also contribute to the L1-induced modulation of neurite outgrowth, cell migration and survival.

### L1-induced increases in FUT9 and ST6Gal1 mRNA levels, neurite outgrowth, cell migration and survival are dependent on phospholipase Cγ

**Figure 5:**
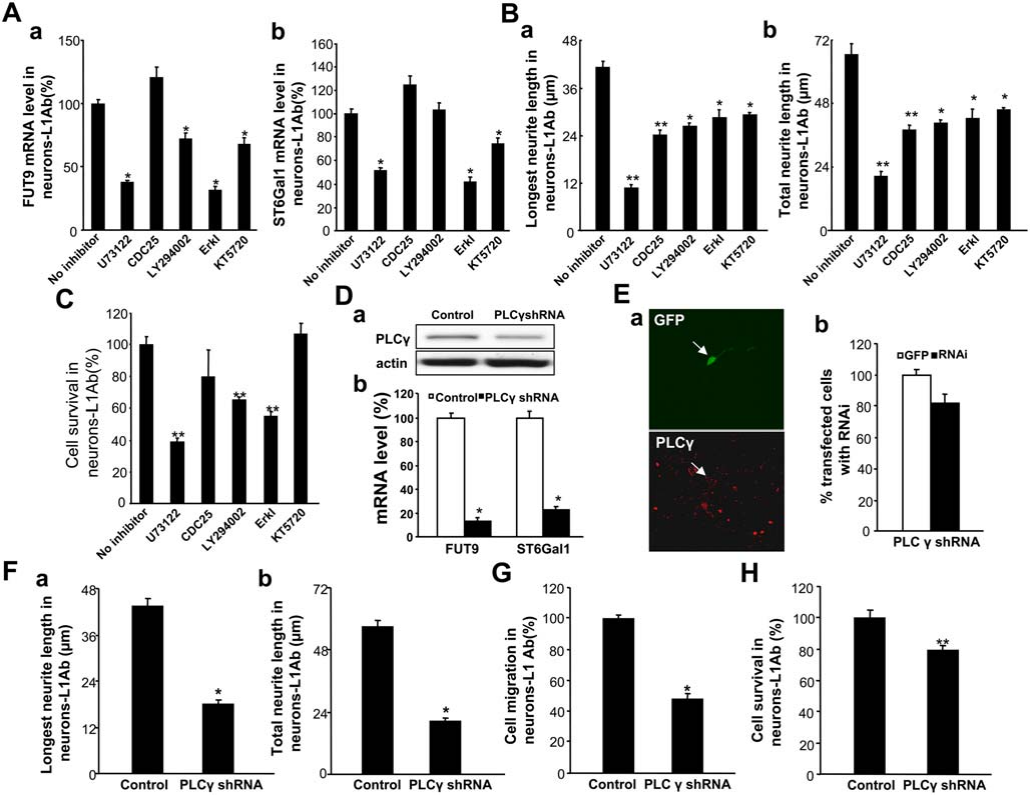
L1 influences FUT9 and ST6Gal1 mRNA levels, neurite outgrowth, cell migration and cell survival via various intracellular signaling mediators. A. Cerebellar granule neurons isolated from the cerebella of wild type mice (6- to 8-day-old) were seeded on coverslips coated with PLL and PLL plus L1 antibodies (neurons-L1Ab). After 1 hour in culture, inhibitors of PLCγ (U73122, 10.5 µM), CDC25 phosphatase (CDC25 inhibitor II, 1.05 µM), PI3K (LY294002, 16.5 µM), Erk (Erk activation inhibitor I, 50 µM) or PKA (KT5720, 280 nM) were added into the culture medium and the cells were cultured for a further 24 hours. Levels of FUT9 mRNA (a) and ST6Gal1 mRNA (b) were quantified by RT-real-time PCR. B. Neurons were seeded on coverslips coated with PLL plus L1 antibodies (L1Ab). Rat IgG coating was used as control. After treatment with the inhibitors of signal transduction pathways listed in A, longest neurite length (a) and total neurite length (b) were measured. C. Neurons were seeded on coverslips coated with PLL plus L1 antibodies (neurons-L1Ab). After treatment with the inhibitors of signal transduction pathways, cell survival was quantified by MTT assay (O.D.: 0.139±0.0065, 0.0538±0.0027, 0.111±0.023, 0.0905±0.0018, 0.0769±0.0035 and 0.148±0.0089, respectively). D. NIH3T3 cells were co-transfected with GFP plasmid plus empty vector (control) or shRNA for PLCγ by electroporation. Twenty four hours later, cells were lysed with RIPA buffer and total protein was obtained. Western blot was used to detect the expression of PLCγ (a). The expression levels of ST6Gal1 and FUT9 mRNAs were detected with real-time PCR after transfected neurons with PLCγ shRNAs or empty vector (control) (b). E. Neurons were co-transfected with GFP plasmid plus empty vector (control) or shRNA for PLCγ by electroporation and seeded on coverslips. After 24 hours in culture, cells were fixed then stained with anti-PLCγ antibodies. (a): Immunocytochemistry for PLCγ (red) in transfected precursors (green) indicated the knockdown efficiency. (b): The co-transfection ratio of GFP and PLCγ shRNA to GFP and empty vector were quantified. F. Neurons were co-transfected with GFP plasmid plus empty vector (control) or shRNA for PLCγ and seeded on coverslips coated with L1 antibody (L1Ab), rat IgG coating was used as control. After 24 hours in culture, the length of the longest neurite length (a) and total neurite length (b) were measured. G. Cell migration assay was performed in L1 antibody-treated neurons (neurons-L1Ab) co-transfected with GFP plasmid plus empty vector (control) or shRNA for PLCγ. The percentage of cell migration was calculated by counting and comparing the number of green neurons on the upper and lower surface of the transwells. H. MTT analysis of L1ab-treated neurons (neurons-L1Ab) was performed after co-transfection with GFP plasmid plus empty vector (control, O.D.: 0.125±0.0054) or shRNA for PLCγ (O.D.: 0.0993±0.0035). *: p<0.05; **: p<0.001, by Student's test, empty plasmid transfection neurons were controls.

To understand the mechanism by which L1 induces changes in sialylation and fucosylation, we investigated the effects of inhibitors of signal transduction pathways known to be activated by L1 on FUT9 and ST6Gal1 mRNA levels. mRNA expression was assayed by RT-real-time PCR. In neurons treated with L1 antibody, the phospholipase Cγ (PLCγ) inhibitor U73122, the PKA inhibitor KT5720 and the Erk inhibitor reduced both FUT9 and ST6Gal1 mRNA levels, with U73122 and Erk inhibitors having the strongest effects (Fig. 5A). The PI3K inhibitor reduced FUT9 mRNA levels but not ST6Gal1 mRNA levels after L1 stimulation. In contrast to this, inhibition of CDC25 had no effect on FUT9 and ST6Gal1 mRNA levels. PLCγ, Cdc25, PI3K, Erk and PKA inhibitors all decreased L1 antibody-induced neurite outgrowth in cerebellar neurons (Fig. 5B), with the strongest effect being produced by the PLCγ inhibitor. PLCγ, PI3K and Erk inhibitors all reduced survival in L1 antibody-treated neurons, whereas the Cdc25 and PKA inhibitors had no effect on survival (Fig. 5C). Since Erk is a downstream element of PLCγ signaling [13], the aforementioned results imply that L1-induced increases in neurite outgrowth may be dependent on the phospholipase Cγ/Erk signaling pathway as was previously shown, e.g. by Chen et al. [29] and Loers et al. [22]. Furthermore, PLCγ shRNA was used to confirm these observations. PLCγ shRNA was transfected into NIH3T cells to confirm that it knocked down tPLCγ expression (Fig. 5Da). Notably, in cerebellar granule neurons treated with L1 antibody, PLCγ shRNA reduced both FUT9 and ST6Gal1 mRNA levels compared with rat IgG treatment (Fig. 5Db). shRNAs of PLCγ significantly reduced the expression of PLCγ in transfected neuronal cells compared with empty vector-transfected cells (Fig. 5E). In neurons treated with L1 antibody, the PLCγ shRNA reduced neurite outgrowth, cell migration and survival when compared with control vector transfection (Fig. 5F, G and H). Thus, taken together these data suggest that L1 is able to regulate sialylation and fucosylation to modulate cell function via a PLCγ dependent pathway.

## Discussion

In our study, we demonstrate that activation of the cell adhesion molecule L1 leads to increased mRNA levels of a brain-relevant sialyltransferase, namely ST6Gal1, and a fucosyltransferase, namely FUT9. This regulation may contribute to the observed changes in sialylation and fucosylation of surface glycoproteins and/or glycolipids in L1-deficient mouse brains. Activation of L1 signal transduction pathways enhances cell survival, migration and neurite outgrowth, which can be blocked by shRNA-mediated knockdown of ST6Gal1 and FUT9. A PLCγ-dependent pathway contributes to the L1-induced increases in expression of the sialyltransferase and fucosyltransferase and to the L1-mediated neurite outgrowth and cell survival.

The central dogma of molecular biology has been revised to include glycosylation or the attachment of glycans or carbohydrates to proteins or lipids, which provides for the needed functional diversity to generate extensive phenotypes from a limited genotype [1]. As glycosylation is not under the direct control of the genome, it is not possible to predict a cell's glycan repertoire from analyses of genomic DNA sequences. Understanding of the importance of glycan function in the nervous and immune systems during development has only recently emerged. Relatively little attention has been paid to the regulation of their expression till now, probably because of their intricate modes of synthesis. Here, we have first shown that the expression of both sialic acids and fucose is significantly reduced at the cell surfaces of *L1^−/y^* neurons, indicating that L1 is able to modulate both sialylation and fucosylation of glycoproteins and/or glycolipids at the surface of neurons. Consistently, we have demonstrated that mRNA levels of the sialyltransferase ST6Gal1 and the fucosyltransferase FUT9 are significantly down-regulated in L1*^−/y^* neurons compared with the levels in L1^+*/y*^ neurons. Moreover, we have shown that stimulation with the L1 antibody 557, which can functionally mimic a L1–L1 *trans*-interaction as described for a scFv antibody [28] recognizing the same domain within the L1 molecule as the 557 antibody, and the stimulation with L1-Fc significantly increased the mRNA levels of the sialyltransferase ST6Gal1 and the fucosyltransferase FUT9 on the cell surfaces of cerebellar granule neurons isolated from *L1^+/y^*, but not *L1^−/y^* mice. This finding indicates that L1–L1 interaction modulated the sialylation and fucosylation on cell surface.

In the nervous system, the diversity of cell–cell interactions is largely mediated by glycans and glycans appear to have been pushed to an even higher degree of sophistication. Some very unusual glycans are pivotal not only during nervous system development and regeneration but also in synaptic plasticity underlying learning and memory [7]. Additionally, glycans contribute to the folding and conformational stability of many proteins [30]–[33] and serve as ligands for glycan-binding proteins that mediate cell trafficking, cell adhesion, and cell signaling [34]–[38]. In agreement with our previous studies [17], [28], we have shown that antibodies to the cell recognition molecule L1, which mimic a L1–L1 *trans*-interaction, enhance neurite outgrowth. Importantly, the anti-L1-antibody-mediated promotion of neurite outgrowth occurs only in neurons isolated from wild-type, but not L1 knockout mice. Since sialylation and fucosylation are enhanced at cell surfaces of the anti-L1 antibody-treated neurons, the specific pattern of glycosylation induced by L1 may also be an important factor in promoting neurite outgrowth and neuronal differentiation. Furthermore, due to the fact that L1 stimulation can modulate sialylation and fucosylation, we have proposed that the L1-induced specific glycosylation pattern may not only influence neurite outgrowth but also contribute, at least partially, to neuronal survival and migration. In agreement with this notion, we have confirmed that L1 promotes cell survival and migration through the L1–L1 trans-interaction mimicked by application of anti-L1 antibody 557, which can be blocked by shRNAs of transferases of sialylation and fucosylation. Thus, these observations demonstrate that L1 may be involved in neuronal survival and migration through its role in modulating glycosylation on cell surface for its specific neuronal functions.

The NCAM and L1 family of adhesion molecules are thought to trigger neurite outgrowth, axonal growth or cell migration by activation of a number of signal transduction pathways, including PLCγ-, PI3K-, Erk- and PKA-dependent pathways [7], [39]. We found that both a PLCγ inhibitor and knockdown of PLCγ by shRNA reduced FUT9 and ST6Gal1 mRNA levels in L1 antibody-treated neurons. Although inhibitors of other signal transduction pathways also had effects on L1-induced neurite outgrowth and cell survival, inhibition of PLCγ consistently resulted in strong inhibition of neurite outgrowth and cell survival in L1 antibody-treated neurons. This suggests that L1 may regulate both sialylation and fucosylation in relation to cell function via a PLCγ-dependent pathway in neurons. Inhibition of PI3K or Erk also reduced L1 antibody-mediated cell survival, as described previously for stimulation with L1-Fc [22]. This may be consistent with the involvement of Erk downstream of PLCγ, but would also be consistent with additional involvement of the Src–PI3K–Erk signaling pathway [7].

During the last decade, increasing evidence has suggested that carbohydrate-carrying molecules in the nervous system have important roles during development, regeneration and synaptic plasticity [7]. In humans, defects in the assembly or processing of N-glycans results in a broad clinical spectrum of inherited disorders, which in most cases show psychomotor and mental retardation or other neurological dysfunctions, such as epilepsy, ataxia, microcephalus, cerebral and cerebellar atrophy, abnormal eye movements and decreased nerve conduction velocity [40]. A successful example of the therapeutic use of a glycan for the treatment of a clinical defect is the oral administration of mannose for the treatment of congenital mannosyl transferase deficiency, a congenital metabolic disease affecting multiple systems, including the nervous system of infants [41]. Here, we have demonstrated that L1 could modulate fucosylation and sialylation of cell surface proteins and/or lipids by regulating the expression of fucosyltransferase 9 and β-galactoside-α2,6-sialyltransferase, which in turn regulates cell survival and migration in neurons. Thus, an important insight of this study is that a distinct glycosylation pattern on the cell surface could be regulated by a specific genetic modulation, which could be applied to manipulate cell-cell communication in development or disease, leading to greater precision and specificity in drug targeting.

## Supporting Information

Figure S1 Western blot result. Cerebellar granule cells from L1+/y and L1−/y mice were lysed and total protein was obtained. Western blotting was used to detect the expression of L1. There was a band at around 200 kDa for the L1+/y neurons and no band for the L1−/y neurons detectable, indicating that L1 is completely knocked out from the L1−/y mice.
(1.85 MB TIF)

Figure S2 Glycosylation patterns on the cell surface of L1+/y neurons, L1−/y neurons, and neurons of L1+/y and L1−/y mice treated with anti-L1 antibodies. Neurons isolated from L1+/y (L1wt) and L1−/y (L1ko) mice and neurons isolated from L1+/y and L1−/y mice treated with anti-L1 antibodies (L1wt-L1Ab and L1ko-L1Ab respectively) were subjected to flow cytometry analysis using a panel of carbohydrate surface markers, including lectins and antibodies against carbohydrates. In the flow cytometry histograms, the filled green areas show the number of unstained cells and the areas outlined in red represent cells binding to various lectins (SNA, MAA, UEAI, DSL, and JAC) and carbohydrates antibody (L5). A. Results of neurons isolated from L1+/y (L1wt) and L1−/y (L1ko) mice. B. Results of neurons from L1+/y and L1−/y mice treated with anti-L1 antibodies (L1wt-L1Ab and L1ko-L1Ab respectively).
(10.39 MB TIF)

Figure S3 L1 promotes neurite outgrowth. Cerebellar granule neurons were isolated from cerebellum of 6- to 8-day-old mice and seeded on coverslips coated with PLL. After 1 hour in culture, L1-Fc fusion protein or Fc (control) was added into the culture medium. The cells were cultured for a further 24 hours and pictures were taken. Neurite length was measured. Left: Longest neurite length. Right: Total neurite length. The results indicate that L1 significantly promotes neurite outgrowth. Data represent mean±SEM of three independent experiments. * p<0.05 significant difference from Fc.
(4.35 MB TIF)

Figure S4 Immunostaining result. Neurons were dissected from the cerebellum of 6- to 8-day-old mice. After 24 hours in culture, cells were fixed and then incubated with anti-SMI-312 antibodies. Immunocytochemistry showed that SMI-312 was positive in neurons. SMI31Ab recognizes the axonal compartment, so this indicates that the measured longest neurites are actually axons.
(7.25 MB TIF)

Figure S5 Cell cycle analysis of neuron cell survival. Cell cycle analysis using propidium iodide (PI) staining was performed as a complimentary experiment to confirm the cell survival. A significant increase in cell survival was observed in L1Ab treated L1+/y neurons (S+G2/M = 34.96%, B) compared with L1+/y neurons treated with rat IgG (control: S+G2/M = 19.23%, A). No such increase was observed in L1−/y neurons treated with L1Ab (S+G2/M = 13.42%, D) compared with L1−/y neurons treated with rat IgG (control: S+G2/M = 10.85%, C). No apoptosis was detected in the cell cultures.
(6.91 MB TIF)
