# Supplementary figures and images for: Cell Surface Sialylation and Fucosylation Are Regulated by L1 via Phospholipase Cγ and Cooperate to Modulate Neurite Outgrowth, Cell Survival and Migration
Source: PLoS One. 2008 Dec 2;3(12):e3841. doi: 10.1371/journal.pone.0003841 (PMC2585790; doi:10.1371/journal.pone.0003841)

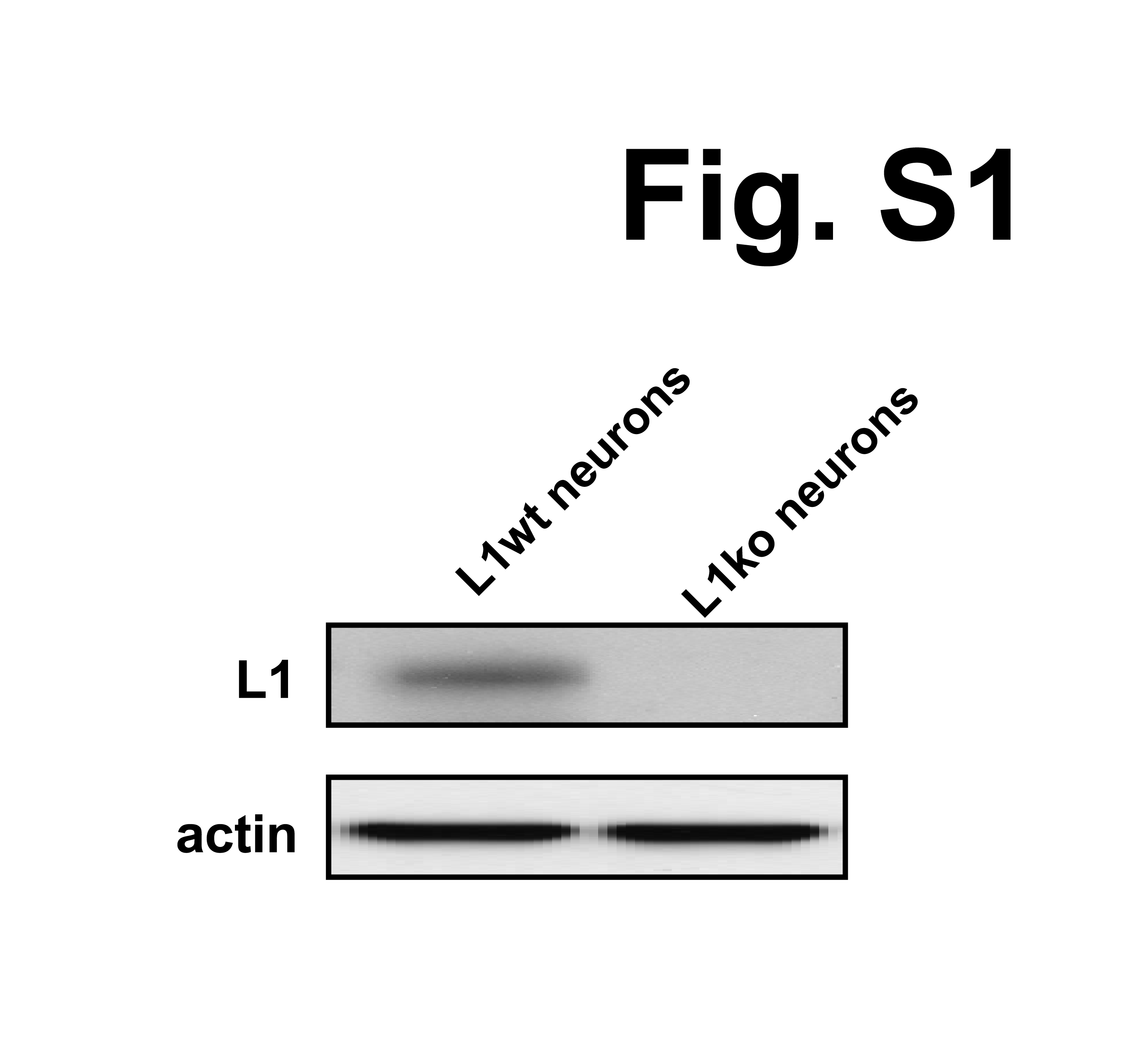

Supplement: Figure S1 — Western blot result. Cerebellar granule cells from L1+/y and L1−/y mice were lysed and total protein was obtained. Western blotting was used to detect the expression of L1. There was a band at around 200 kDa for the L1+/y neurons and no band for the L1−/y neurons detectable, indicating that L1 is completely knocked out from the L1−/y mice. (1.85 MB TIF) [file pone.0003841.s001.tif]

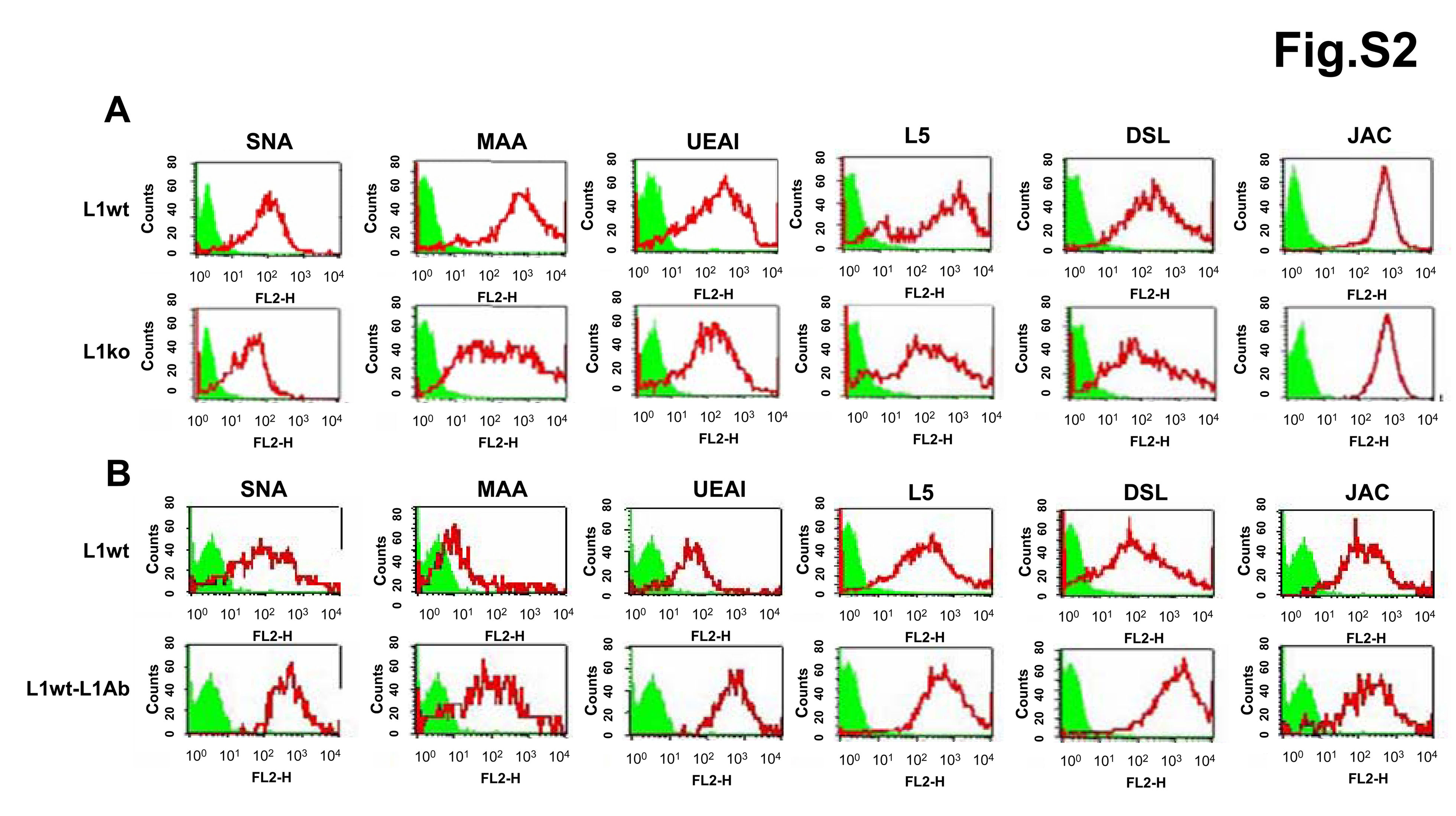

Supplement: Figure S2 — Glycosylation patterns on the cell surface of L1+/y neurons, L1−/y neurons, and neurons of L1+/y and L1−/y mice treated with anti-L1 antibodies. Neurons isolated from L1+/y (L1wt) and L1−/y (L1ko) mice and neurons isolated from L1+/y and L1−/y mice treated with anti-L1 antibodies (L1wt-L1Ab and L1ko-L1Ab respectively) were subjected to flow cytometry analysis using a panel of carbohydrate surface markers, including lectins and antibodies against carbohydrates. In the flow cytometry histograms, the filled green areas show the number of unstained cells and the areas outlined in red represent cells binding to various lectins (SNA, MAA, UEAI, DSL, and JAC) and carbohydrates antibody (L5). A. Results of neurons isolated from L1+/y (L1wt) and L1−/y (L1ko) mice. B. Results of neurons from L1+/y and L1−/y mice treated with anti-L1 antibodies (L1wt-L1Ab and L1ko-L1Ab respectively). (10.39 MB TIF) [file pone.0003841.s002.tif]

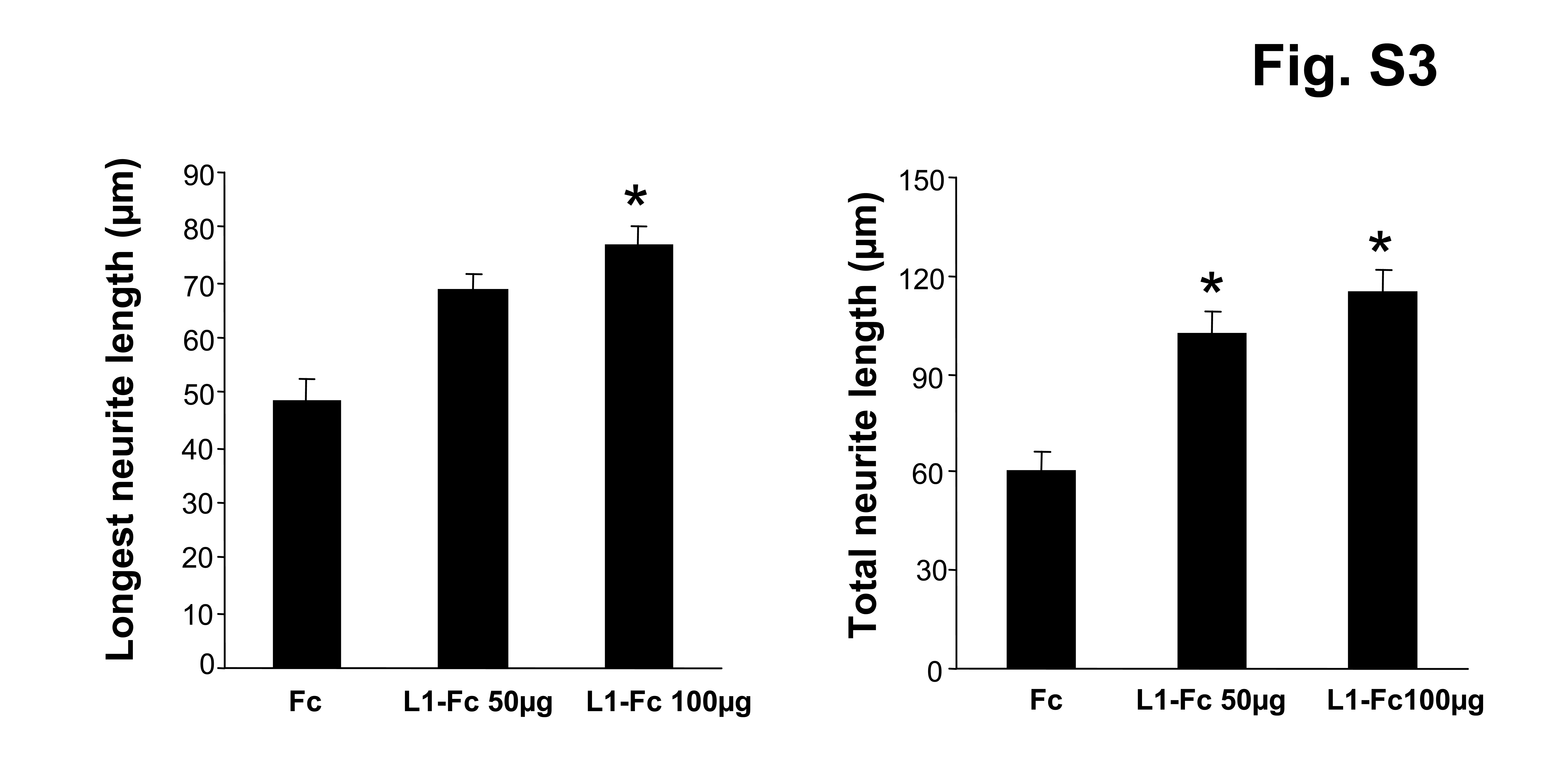

Supplement: Figure S3 — L1 promotes neurite outgrowth. Cerebellar granule neurons were isolated from cerebellum of 6- to 8-day-old mice and seeded on coverslips coated with PLL. After 1 hour in culture, L1-Fc fusion protein or Fc (control) was added into the culture medium. The cells were cultured for a further 24 hours and pictures were taken. Neurite length was measured. Left: Longest neurite length. Right: Total neurite length. The results indicate that L1 significantly promotes neurite outgrowth. Data represent mean±SEM of three independent experiments. * p<0.05 significant difference from Fc. (4.35 MB TIF) [file pone.0003841.s003.tif]

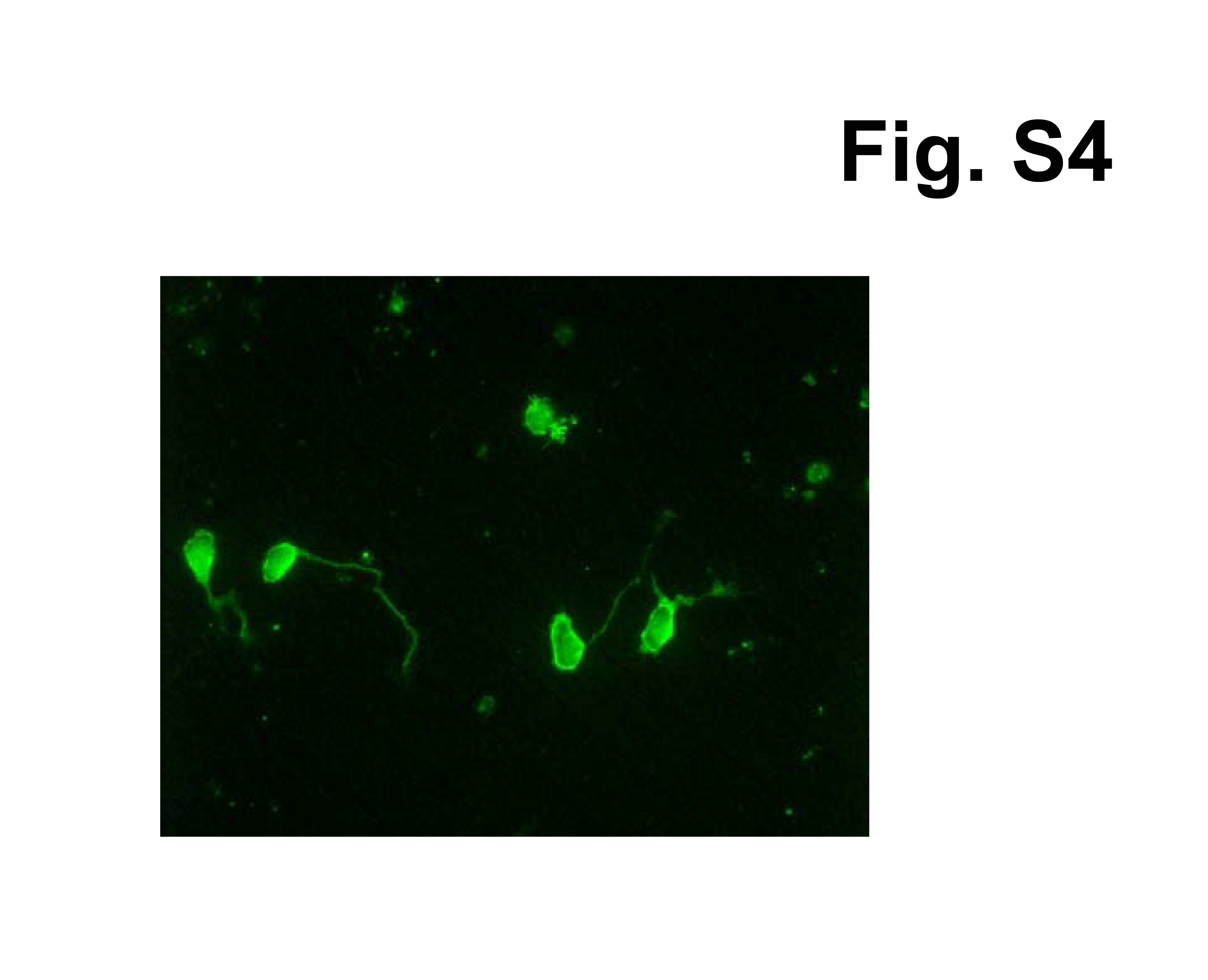

Supplement: Figure S4 — Immunostaining result. Neurons were dissected from the cerebellum of 6- to 8-day-old mice. After 24 hours in culture, cells were fixed and then incubated with anti-SMI-312 antibodies. Immunocytochemistry showed that SMI-312 was positive in neurons. SMI31Ab recognizes the axonal compartment, so this indicates that the measured longest neurites are actually axons. (7.25 MB TIF) [file pone.0003841.s004.tif]

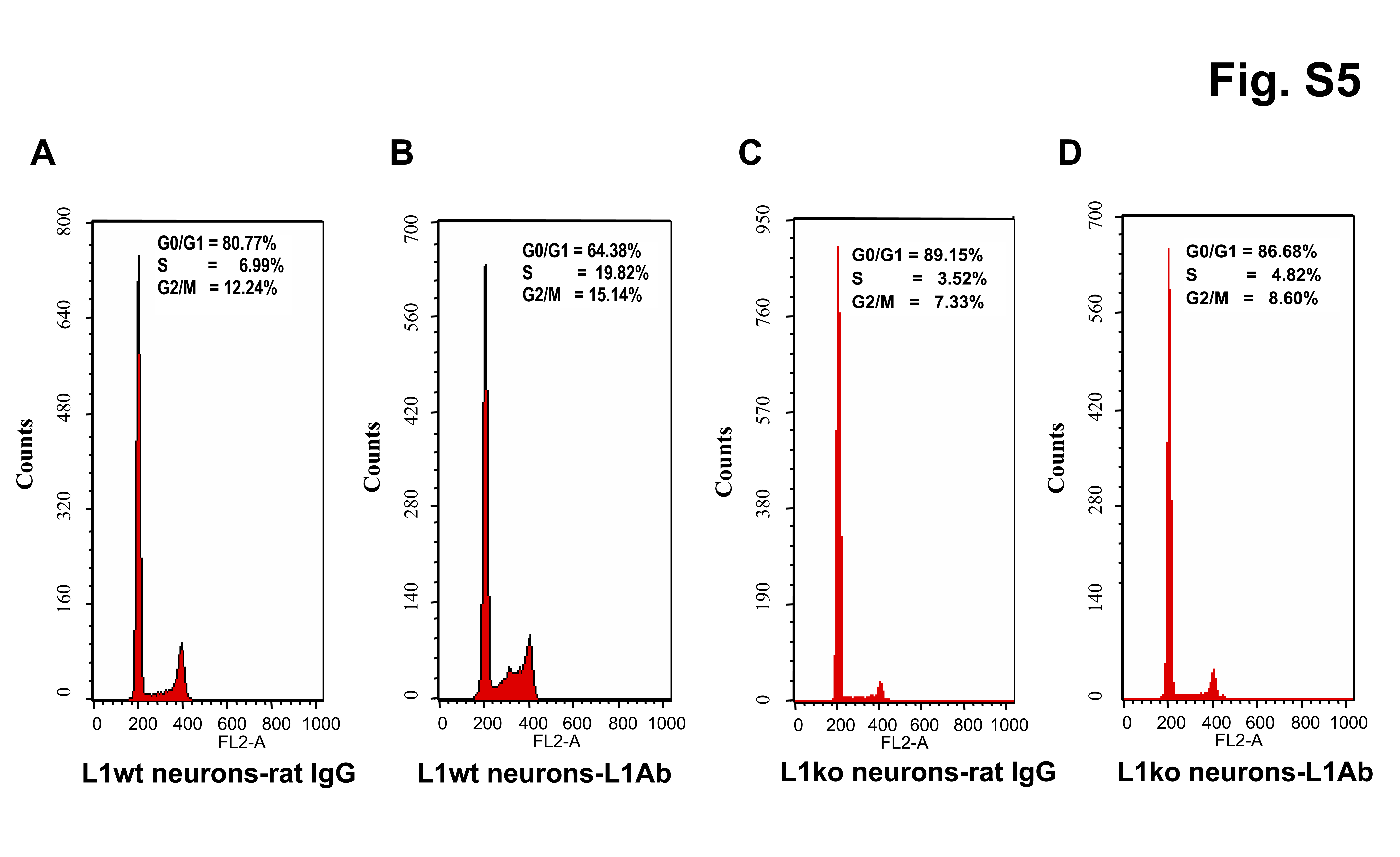

Supplement: Figure S5 — Cell cycle analysis of neuron cell survival. Cell cycle analysis using propidium iodide (PI) staining was performed as a complimentary experiment to confirm the cell survival. A significant increase in cell survival was observed in L1Ab treated L1+/y neurons (S+G2/M = 34.96%, B) compared with L1+/y neurons treated with rat IgG (control: S+G2/M = 19.23%, A). No such increase was observed in L1−/y neurons treated with L1Ab (S+G2/M = 13.42%, D) compared with L1−/y neurons treated with rat IgG (control: S+G2/M = 10.85%, C). No apoptosis was detected in the cell cultures. (6.91 MB TIF) [file pone.0003841.s005.tif]
